# Simvastatin Enhances Activity and Trafficking of α7 Nicotinic Acetylcholine Receptor in Hippocampal Neurons Through PKC and CaMKII Signaling Pathways

**DOI:** 10.3389/fphar.2018.00362

**Published:** 2018-04-12

**Authors:** Tingting Chen, Ya Wang, Tingting Zhang, Baofeng Zhang, Lei Chen, Liandong Zhao, Ling Chen

**Affiliations:** ^1^State Key Laboratory of Reproductive Medicine, Nanjing Medical University, Nanjing, China; ^2^Department of Physiology, Nanjing Medical University, Nanjing, China; ^3^Department of Neurology, Huaian Second People’s Hospital, Huaian, China

**Keywords:** simvastatin, α7 nicotinic acetylcholine receptor (α7nAChR), farnesyl pyrophosphate (FPP), farnesyl transferase inhibitor (FTI), protein kinas C (PKC), calmodulin-kinase II (CaMKII)

## Abstract

Simvastatin (SV) enhances glutamate release and synaptic plasticity in hippocampal CA1 region upon activation of α7 nicotinic acetylcholine receptor (α7nAChR). In this study, we examined the effects of SV on the functional activity of α7nAChR on CA1 pyramidal cells using patch-clamp recording and explored the underlying mechanisms. We found that the treatment of hippocampal slices with SV for 2 h induced a dose-dependent increase in the amplitude of ACh-evoked inward currents (*I*_ACh_) and the level of α7nAChR protein on the cell membrane without change in the level of α7nAChR phosphorylation. These SV-induced phenotypes were suppressed by addition of farnesol (FOH) that converts farnesyl pyrophosphate, but not geranylgeraniol. Similarly, the farnesyl transferase inhibitor FTI277 was able to increase the amplitude of *I*_ACh_ and enhance the trafficking of α7nAChR. The treatment with SV enhanced phosphorylation of CaMKII and PKC. The SV-enhanced phosphorylation of CaMKII rather than PKC was blocked by FOH, Src inhibitor PP2 or NMDA receptor antagonist MK801 and mimicked by FTI. The SV-enhanced phosphorylation of PKC was sensitive to the IP3R antagonist 2-APB. The SV-increased amplitude of *I*_ACh_ was suppressed by PKC inhibitor GF109203X and Go6983, or CaMKII inhibitor KN93. The SV- and FTI-enhanced trafficking of α7nAChR was sensitive to KN93, but not GF109203X or Go6983. The PKC activator PMA increased α7nAChR activity, but had no effect on trafficking of α7nAChR. Collectively, these results indicate that acute treatment with SV enhances the activity and trafficking of α7nAChR by increasing PKC phosphorylation and reducing farnesyl-pyrophosphate to trigger NMDA receptor-mediated CaMKII activation.

## Introduction

Statins, which are inhibitors of 3-hydroxy-3-methyl-glytarylcoenzyme A (HMA-CoA) reductase, have received much attention due to the lower prevalence of Alzheimer’s disease (AD) and/or dementia in patients treated with these drugs (Wolozin et al., 2000). In a 26-week randomized placebo-controlled double-blind trial, treatment with simvastatin (SV) improved cognitive functions in normal cholesterolemic AD patients (Simons et al., 2002). Treatment with SV can improve cognition in AD patients through a mechanism unrelated to its cholesterol-lowering effects (Jick et al., 2000).

Pathological studies on the brains of humans with AD revealed α7 nicotinic acetylcholine receptor (α7nAChR) localizes in neuritic plaques (Wang et al., 2000). The decline of α7nAChR level and dysfunction of α7nAChR have been found in the brains of AD patients (Burghaus et al., 2000; Kihara et al., 2004). One important characteristic of α7nAChR is its high permeability to Ca^2+^, thus α7nAChR plays an important role in synaptic plasticity and cognitive function (Sharma and Vijayaraghavan, 2003; Chen et al., 2016a). The dysfunction of α7nAChR is known to be critical pathogenic process and mechanism of cognitive disorder in AD (Chen et al., 2006). The coapplication of mevastatin and myriocin has been reported to alter the desensitization kinetics of α7nAChR in the membrane (Colon-Saez and Yakel, 2011). The chronic administration of SV in mice enhances the presynaptic glutamate release and facilitates the hippocampal long-term potentiation induction via the activation of α7nAChR (Chen et al., 2016a).

Statins not only reduce *de novo* cholesterol biosynthesis, but also prevent the conversion of 3-hydroxy-3-methyl-glytarylcoenzyme A into mevalonate, leading to reduction of non-sterol intermediates known as isoprenoids (Endo, 2004). Two types of isoprenoids, farnesyl-pyrophosphate (FPP) and geranylgeranyl-pyrophosphate (GGPP), are essential for the prenylation of small GTP-binding proteins (GTPases) (McTaggart, 2006). The treatment with SV results in a reduction in FPP level followed by a decline in GGPP and cholesterol levels (Eckert et al., 2009). The chronic or acute administration of SV can enhance the induction of long-term potentiation through the reduction of FPP and inhibition of farnesylation (Mans et al., 2012; Chen et al., 2016a). The farnesylated proteins include the Ras superfamily of GTPases (e.g., H-Ras, K-Ras, and N-Ras) (Kho et al., 2004; McTaggart, 2006). The activation of Ras modifies downstream effectors protein kinase C (PKC) (Dann et al., 2014) and Src (Thornton et al., 2003). Phosphorylation of PKC can modulate the Ca^2+^ channels of α7nAChR (Huganir and Greengard, 1990). We have recently reported that the administration of SV can increase Src phosphorylation leading to augmentation of NMDAr activity (Chen et al., 2016b). Ca^2+/^calmodulin-dependent protein kinase II (CaMKII) is activated in hippocampal neurons by the increasing Ca^2+^ influx through NMDAr channels. The activating CaMKII is able to enhance the response of α7nAChR (Kanno et al., 2012a). In addition, Serine 365 in the M3-M4 cytoplasmic loop of the α7nAChR is a phosphorylation site for protein kinase A (PKA) (Moss et al., 1996). The disruption of lipid rafts affects the localization and mobility of α7nAChR (Oshikawa et al., 2003). Collectively, we hypothesized that the acute treatment with SV is able to regulate the activity and trafficking of α7nAChR.

In the present study, we focused on the acute roles of SV and farnesyl transferase inhibitor (FTI) in the activity, trafficking and phosphorylation of α7nAChRs in hippocampal CA1 pyramidal cells. We further examined the effects of SV and FTI on PKC-, PKA-, and CaMKII-signaling pathways and explored the underlying mechanisms of SV-altered α7nAChR activity and trafficking. Our results in this study indicate that acute treatment with SV enhances the activity and trafficking of α7nAChR by increasing phosphorylation of PKC and reducing FPP to promote the CaMKII signaling pathway.

## Materials and Methods

### Experimental Animals

The present study was approved by Animal Care and Ethical Committee of Nanjing Medical University. All animal handling procedures followed the guidelines of Institute for Laboratory Animal Research of the Nanjing Medical University. The procedures involving animals and their care were conducted in conformity with the ARRIVE guidelines of Laboratory Animal Care (Kilkenny et al., 2012). Postnatal 28–32 days male mice (ICR, Oriental Bio Service Inc., Nanjing) were used. The animals were maintained in a constant environmental condition (temperature 23 ± 2°C, humidity 55 ± 5%, 12:12 h light/dark cycle) in the Animal Research Center of Nanjing Medical University. They had free access to food and water.

### Preparation and Administration of Drugs

Simvastatin (Enzo Life Sciences International, Farmingdale, NY, United States) was converted from its inactive lactone prodrug form to its active dihydroxy open acid form by alkaline hydrolysis (Mans et al., 2010). SV (50 mg) was dissolved in 1 ml ethanol (100%), and then added by 0.813 ml NaOH (l N), and the resulting solution was stored in aliquots at -20°C (for up to 1 month). The stock solution was neutralized with l N HC1 to pH of 7.4 and diluted in artificial cerebral spinal fluid (ACSF).

Trans, trans-farnesol (FOH, 96%, Cat# 277541) and Geranylgeraniol (GGOH, 85%, Cat# G3278) were purchased from Sigma (St. Louis, MO, United States). FOH and GGOH were initially pipetted into ethanol, and then diluted by ACSF to final concentration of 0.01% ethanol.

Farnesyl transferase inhibitor (FTI, FTI-277) was purchased from Calbiochem (Cat# 344555) (Darmstadt, Germany). FTI (250 μg) was dissolved in 559 μl dimethyl sulfoxide (DMSO) to prepare the stock solution at 1 mM concentration.

The α7nAChR agonist acetylcholine (ACh), α7nAChR antagonist methyl-lycaconitine (MLA), NMDA inhibitor MK801, Src inhibitor PP2, PKC inhibitor GF109203X (GF) and Go6983 (Go), PKC agonist phorbol 12-myristate 13-acetate (PMA) and inositol-1,4,5-trisphosphate receptor (IP3R) antagonist 2-APB were purchased from Sigma (St. Louis, MO, United States), PKA inhibitor H89 and CaMKII inhibitor KN93 were purchased from Medchem express (NJ, United States). The drugs were dissolved in DMSO and diluted by ACSF to a final 0.1% concentration of DMSO.

### Hippocampal Slices Preparation

The mice were deeply anesthetized with isoflurane and decapitated. The brains were rapidly removed and coronal brain slices (400 μm) were cut using a vibrating microtome (Microslicer DTK 1500, Dousaka EM Co, Kyoto, Japan) in ice-cold cutting solution (in mM: 94 sucrose, 30 NaCl, 4.5 KCl, 1 MgCl_2_, 26 NaHCO_3_, 1.2 NaH_2_PO_4_, and 10 D-glucose, pH 7.4) oxygenated with a gas mixture of 95% O_2_/5% CO_2_. The hippocampal slices were then incubated in ACSF (in mM: 126 NaCl, 1 CaCl_2_, 2.5 KCl, 1 MgCl_2_, 26 NaHCO_3_, 1.25 KH_2_PO_4_, and 20 D-glucose, pH 7.4) oxygenated with a gas mixture of 95% O_2_/5% CO_2_ at 32–34°C.

### Electrophysiological Analysis

After 1 h recovery, the slices were transferred to a recording chamber for whole cell patch-clamp recording. The slice was perfused continually with oxygenated ACSF. The glass pipette (4–5 MΩ resistance) was filled with an internal solution (in mM: Cs-gluconate 120, NaCl 2, MgCl_2_ 4, Na_2_-ATP 4, HEPES 10, and EGTA 10) at pH 7.2. Atropine (0.5 mM) was added to the external solution to block muscarinic acetylcholine receptors. In addition, 10 μM bicuculline, 20 μM AP-5, 10 μM NBQX, 0.1 μM TTX were applied extracellularly. The holding potential was -70 mV. The α7nAChR-activated current (*I*_ACh_) was induced by the application of ACh using a rapid drug delivery system as described (Colon-Saez and Yakel, 2011; Li et al., 2013) and recorded using an EPC-10 amplifier (HEKA Elektronik, Lambrecht/Pfalz, Germany). Peak currents, decay kinetics, and curve fitting were measured and analyzed using Clampfit (Molecular Devices) Origin (OriginLab Corp., Northampton, MA, United States), and Sigmaplot10. The *I*_ACh_ was normalized to *I*_ACh_ evoked by 3 mM ACh in the same neuron to produce dose-response curve. The data were fitted to Logistic equation in which *I* = *I*max/[1+(EC_50_/*C*)*^n^*], with *n* being Hill coefficient and EC_50_ being the concentration producing 50% maximal response. The α7nAChRs desensitization was calculated by the half-time of desensitization that was required for 50% decay of peak *I*_ACh_ amplitude (Gay et al., 2008).

### Slices Biotinylation

After 1 h recovery, the hippocampal slices were washed once for 5 min in ice-cold ACSF, and then incubated with ACSF containing EZ-link Sulfo-NHS-SS-Biotin (0.5 mg/ml, Pierce, Northumberland, United Kingdom) on a shaker for 25 min at 4°C. To remove excess biotin, slices were washed three times with 50 mM NH_4_Cl in ACSF for 5 min at 4°C. The hippocampal tissue was dissected out and homogenized in lysis buffer containing 50 mM Tris–HCl (pH 7.4), 150 mM NaCl, 1.5 mM MgCl_2_, 1 mM EGTA, 0.5 mM DTT, 50 mM NaF, 2 mM sodium pyruvate, 25% glycerol, 1% triton X-100, 0.5% sodium deoxycholate, and 1% protease inhibitor cocktail (Sigma). Following the centrifugation at 20,000 × *g* for 20 min at 4°C, the supernatants were collected as the source of proteins. The protein concentration was determined by Bradford protein assay. The biotinylated proteins (50 μg) were incubated with streptavidin-coated magnetic beads (30 μl) on a head-over-head shaker for 45 min at room temperature. The streptavidin beads attaching biotinylated proteins were washed three times with lysis buffer containing 0.1% SDS, and separated by a magnet. The biotinylated proteins were eluted in sample buffer (62.5 mM Tris–HCl, 2% SDS, 5% glycerol, 5% 2-mercaptoethanol) at 100°C for 5 min. The protein lysates were also denatured in the same method. The protein lysates (cytoplasmic proteins) and biotinylated proteins (cell surface proteins) were frozen until analysis.

### Immunoprecipitation and Western Blot Analysis

The mice were deeply anesthetized with isoflurane and decapitated. The hippocampus were rapidly removed and homogenized in a lysis buffer containing 50 mM Tris–HCl (pH 7.5), 150 mM NaCl, 5 mM EDTA, 10 mM NaF, 1 mM sodium orthovanadate, 1% Triton X-100, 0.5% sodium deoxycholate, 1 mM phenylmethylsulfonyl fluoride and protease inhibitor cocktail (Complete; Roche, Mannheim, Germany). The incubations were performed for 30 min at 4°C with shaking. Protein concentration was determined with BCA Protein Assay Kit (Pierce Biotechnology Inc., Rockford, IL, United States).

For immunoprecipitation assays, total proteins (500 μg) were incubated with rabbit anti-α7nAChR antibody (1:1000; Chemicon, CA, United States) overnight at 4°C. Next, 40 μl protein A/G-Sepharose (GE Healthcare, Sweden) was added and the samples were incubated for 1 h at 4°C in a shaker. The immunocomplexes were centrifuged at 4°C for 5 min at 1000 × g and washed four times with homogenization buffer (Contreras-Vallejos et al., 2014). The supernatants were analyzed by Western blotting.

For Western blot, total proteins (20 μg) were separated by SDS-polyacrylamide gel electrophoresis (SDS–PAGE) and transferred to a polyvinylidene fluoride (PVDF) membrane. The membranes were incubated with antibodies of rabbit polyclonal anti-α7nAChR (1:1000; Abcam, Cambridge, United Kingdom), anti-phospho-PKA (1:1000; Millipore, MA, United States), anti-phospho-PKC (1:1000; Abcam, Cambridge, United Kingdom), anti-phospho-CaMKII (1:1000; Cell Signaling Technology, MA, United States), anti-phospho-Ser (1:1000; Santa Cruz, CA, United States), and anti-phospho-Thr (1:1000; Santa Cruz, CA, United States). After washing, the membranes were incubated with horse radish peroxidase-labeled goat anti-rabbit antibody (1:5000; Santa Cruz, CA, United States), and developed using the enhanced chemiluminescence detection Kit (Millipore). Following visualization, the blots were stripped by incubation in stripping buffer (Restore; Pierce) for 15 min, and then incubated with antibodies of anti-PKC (1:1000; Abcam), anti-PKA (1:1000; Millipore) and anti-CaMKII (1:1000; Abcam), or anti-β-actin (1:1000, Cell Signaling Technology). Western blot bands were scanned and analyzed with the image analysis software package (Image J; NIH Image, Bethesda, MD, United States). AMPA receptor subunits glutamic acid receptor 2 (GluR2) was highly expressed in the membrane surface of hippocampal neuronal cells (Mielke and Mealing, 2009). We observed that the treatment of slices with SV (10 μM) for 2 or 4 h had no effect on the AMPA receptor activity (Chen et al., 2016b) and the levels of surface GluR2 protein (**Figure 2A**). Thus, in this study the biotinylated membrane surface α7nAChR protein was normalized by surface GluR2 protein and cytoplasmic α7nAChR protein was normalized by β-actin.

### Data Analysis

The group data were expressed as the means ± standard error (SE). All statistical analyses were performed using SPSS software, version 16.0 (SPSS Inc., United States). Differences among means were analyzed using the Student’s *t*-tests or analyses of variance (ANOVA) with or without repeated measures, followed by Bonferroni *post hoc* analysis. Differences at *P* < 0.05 were considered statistically significant.

## Results

### Effects of SV on α7nAChR Activity in Hippocampal CA1 Pyramidal Cells

**FIGURE 1:**
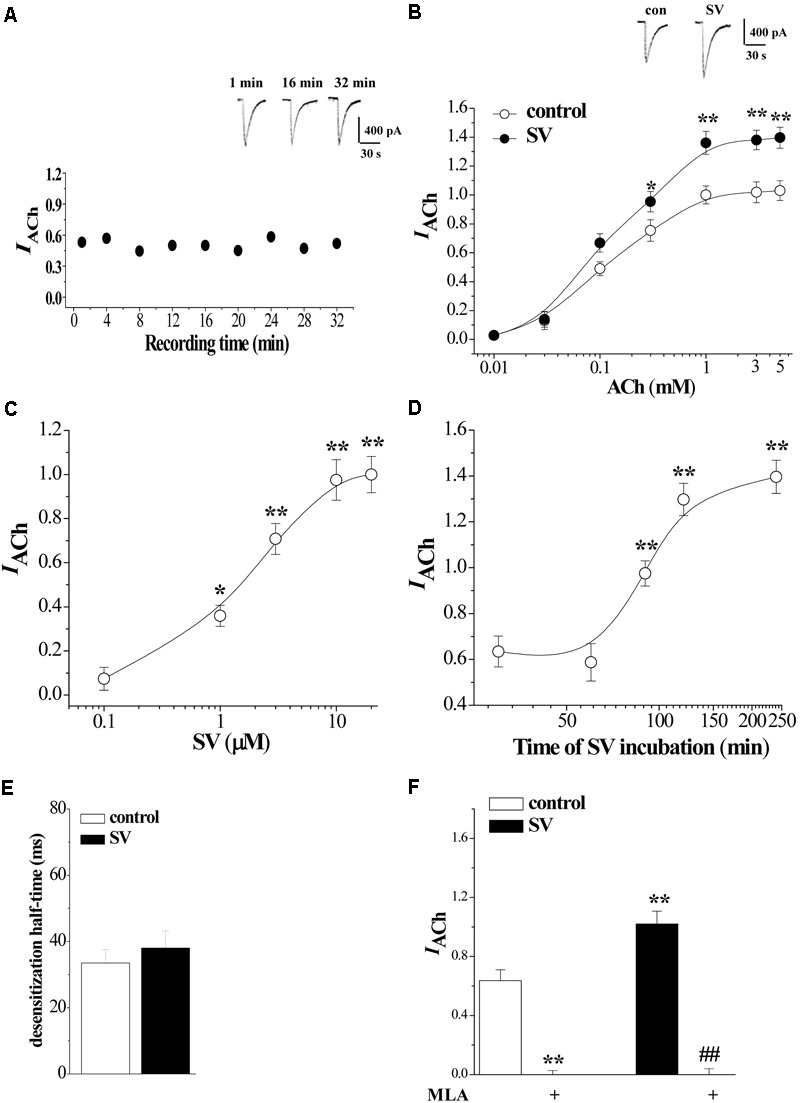
Acute treatment with Simvastatin enhances α7nAChR activity in hippocampal CA1 pyramidal cells. **(A)** Repeated applications of ACh (1.0 mM) with 4 min interval evoke whole cell currents (*I*_ACh_). Representative traces of *I*_ACh_ evoked at the time of 1, 16, and 32 min. **(B)** The CA1 pyramidal cells were subjected to consecutive 1 sec applications of 0.01, 0.03, 0.1, 0.3, 1, 3, and 5 mM ACh. Dose-response curves were constructed by the amplitude of *I*_ACh_ (means ± SEM) that were normalized by a control value evoked by ACh (3 mM). Representative traces of *I*_ACh_ evoked by 1 mM ACh. ^∗^*P* < 0.05 and ^∗∗^*P* < 0.01 vs. control slices (repeated-measure ANOVA). **(C)** Evoked *I*_ACh_ by ACh (1 mM) in the slices treated with SV at 0.1, 1, 3, 10, and 20 μM for 2 h. Dose-response curves were constructed by the amplitude of *I*_ACh_ (means ± SEM) that were normalized by the treatment with 10 μM SV. ^∗^*p* < 0.05 and ^∗∗^*p* < 0.01 vs. vehicle-treated control (repeated-measure ANOVA). **(D)** Evoked *I*_ACh_ by ACh (1 mM) in the slices treated with SV (10 μM) for 30, 60, 90, 120, and 240 min. Time-dependent curves were constructed by the amplitude of *I*_ACh_ (means ± SEM). ^∗∗^*p* < 0.01 vs. vehicle-treated control (repeated-measure ANOVA). **(E)** Influence of SV treatment (10 μM, 2 h) on the desensitization half-time of α7nAChR. **(F)** Sensitivity of evoked *I*_ACh_ to α7nAChR antagonist MLA. Mean percent reduction in the amplitude of *I*_ACh_ evoked by ACh (1 mM) following application of MLA (10 μM) in control slices and SV-treated slices. ^∗∗^*p* < 0.01 vs. control slices; ^##^*p* < 0.01 vs. SV-treated slices (two-way ANOVA).

ACh-evoked inward current (*I*_ACh_) in hippocampal CA1 pyramidal cells was examined using whole cell patch-clamp recording. The α7nAChRs completely recovered from desensitization after washing for 1 min with ACh (Colon-Saez and Yakel, 2011). During repeated ejections of ACh (1 mM) at 4 min intervals, the *I*_ACh_ densities remained stable and exhibited no rundown for 30–40 min (*n* = 7–8 cells/4 mice per experimental group; **Figure 1A**). ACh dose-response curves were constructed to evaluate the response of α7nAChR to different concentrations of ACh (**Figure 1B**), where *I*_ACh_ amplitude displayed a concentration-dependent increase (*F*_6,49_ = 80.407, *p* < 0.001).

To investigate the acute effect of SV on α7nAChR activity, hippocampal slices (400 μm) were incubated in 0.1–20 μM concentrations of SV for 2 h or treated with 10 μM SV for 0.5–4 h. The SV treatment does not alter the intrinsic properties of pyramidal cell membranes (Parent et al., 2014). As shown in **Figure 1C**, the treatment with SV could increase the amplitude of *I*_ACh_ in a dose-dependent manner (*F*_4,35_ = 58.465, *p* < 0.001). In addition, the SV-induced increases in *I*_ACh_ amplitude required treatment to last for longer than 90 min (*F*_4,35_ = 30.806, *p* < 0.001; **Figure 1D**).

According to the above results, we examined the influence of SV (10 μM)-treatment for 2 h in the sensitivity of α7nAChR to agonists. As shown in **Figure 1B**, the treatment of SV altered the dose-response curves of *I*_ACh_ (*F*_6,49_ = 75.836, *p* < 0.001). In comparison with controls, the treatment with SV significantly increased the *I*_ACh_ amplitude (*F*_1,14_ = 25.035, *p* < 0.001). The value of EC_50_ (124.0 ± 1.67 μM) and Hill coefficient (1.50) did not differ significantly from controls (EC_50_ = 117.1 ± 0.94 μM; Hill coefficient = 1.44; *p* > 0.05). In addition, the SV treatment did not cause the change in the desensitization half-time (ms) of *I*_ACh_ (*p* > 0.05; **Figure 1E**). The SV-induced increase in *I*_ACh_ amplitude was sensitive to the perfusion of α7nAChR antagonist MLA (10 μM) for 5 min (*p* < 0.01; **Figure 1F**). The results show that the treatment with SV (>1 μM) for over 90 min can potentiate the α7nAChR activity without changes in the agonist sensitivity and the kinetics of desensitization.

### Effects of SV on the Distribution of α7nAChRs on Hippocampal CA1 Neurons

**FIGURE 2:**
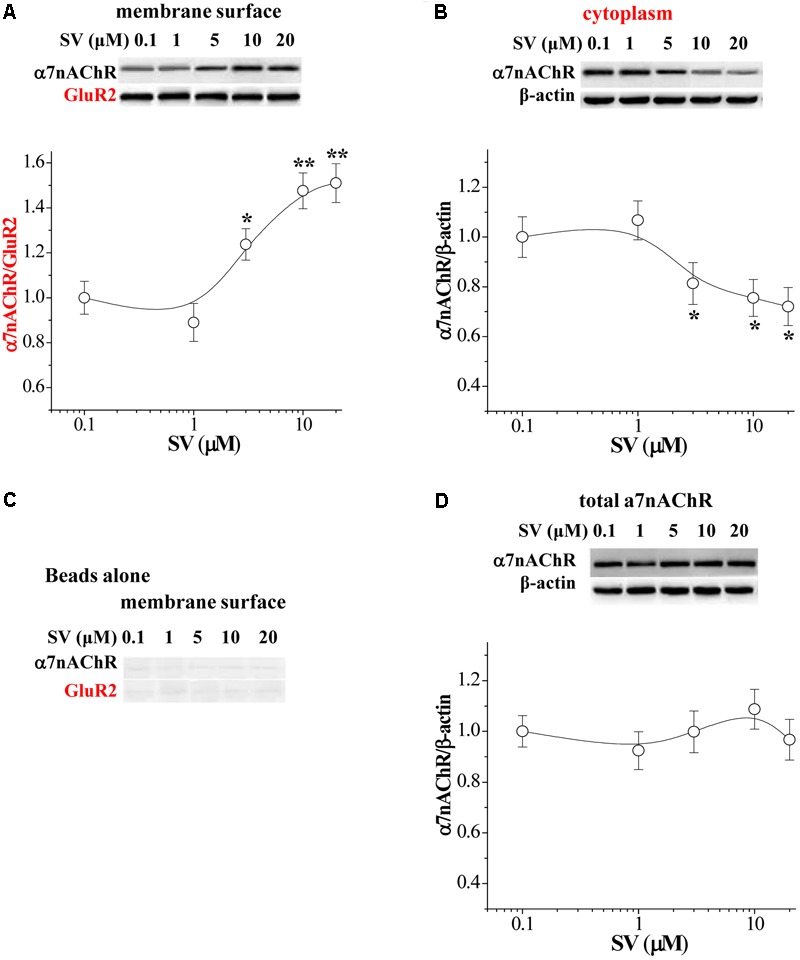
Acute treatment of SV enhances the trafficking of α7nAChR. **(A,B)** Representative Western blots of cell membrane surface (surface) and cytoplasmic α7nAChR protein in hippocampus. Biotin/beads: biotinylated samples. Graphs indicate the mean levels of surface α7nAChR **(A)** and cytoplasmic α7nAChR **(B)** in control and SV-treated hippocampus. Surface α7nAChR was normalized by surface GluR2 protein and cytoplasmic α7nAChR was normalized by β-actin, which was again normalized by vehicle-treated group. ^∗^*p* < 0.05 and ^∗∗^*p* < 0.01 vs. control slices (two-way ANOVA). **(C)** “Beads alone” indicates non-biotinylated samples as a negative control. **(D)** Total α7nAChR protein was normalized by β-actin, which was again normalized by vehicle-treated group.

The enhanced membrane surface localization of α7nAChR can increase α7nAChR response (Kanno et al., 2012a,b). To assess localization of α7nAChRs, we measured the levels of biotinylated α7nAChR protein and cytoplasmic α7nAChR protein in the hippocampus (*n* = 6 mice per experimental group; 3–4 slices/mouse were treated), respectively. Western blot analysis revealed an increase in the level of biotinylated α7nAChR protein that was dependent on the concentrations of SV (0.1–20 μM) (*F*_4,25_ = 7.607, *p* < 0.001; **Figure 2A**), which was associated with a dose-dependent decrease in the level of cytoplasmic α7nAChR protein (*F*_4,25_ = 3.869, *p* = 0.014; **Figure 2B**). By contrast, the levels of non-biotinylated membrane (**Figure 2C**) and total α7nAChR protein were not altered by the treatment with SV (*F*_4,25_ = 0.516, *p* = 0.724; **Figure 2D**). The results show that the treatment with SV is able to enhance the trafficking of α7nAChR.

### Involvement of SV-Reduced Isoprenoids in α7nAChR Activity and Trafficking

**FIGURE 3:**
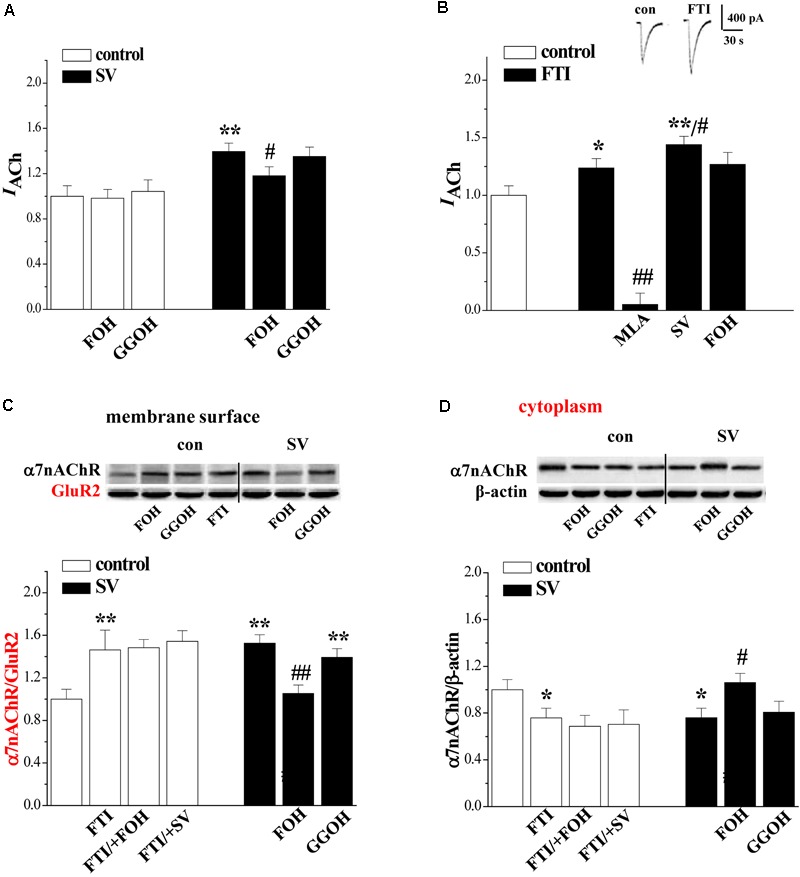
Simvastatin through reducing FPP enhances α7nAChR activity and trafficking. **(A)** Effects of FOH or GGOH addition in SV-enhanced α7nAChR activity. Bars show the amplitude of *I*_ACh_ in control slices, SV-treated slices, SV/+FOH- and SV/+GGOH-treated slices. ^∗∗^*p* < 0.01 vs. control slices and ^#^*p* < 0.05 vs. SV-treated slices (two-way ANOVA). **(B)** Effects of FTI on α7nAChR activity in hippocampal CA1 pyramidal cells. Bars show the amplitude of *I*_ACh_ in FTI/+MLA-, FTI/+SV- and FTI/+FOH-treated slices. ^∗^*p* < 0.05 and ^∗∗^*p* < 0.01 vs. control slices and ^#^*p* < 0.05 and ^##^*p* < 0.01 vs. FTI-treated slices (two-way ANOVA). **(C,D)** Effects of FOH or GGOH addition in SV-enhanced α7nAChR trafficking. Bar graphs show the levels of biotinylated α7nAChR protein and cytoplasmic α7nAChR protein in control slices, FTI-treated slices, FTI/+FOH- and FTI/+SV-treated slices, SV/+FOH- or SV/+GGOH-treated slices. Surface α7nAChR was normalized by surface GluR2 protein and cytoplasmic α7nAChR was normalized by β-actin.^∗^*p* < 0.05 and ^∗∗^*p* < 0.01 vs. control slices. ^#^*p* < 0.05 and ^##^*p* < 0.01 vs. SV-treated slices (two-way ANOVA).

To test whether SV enhances α7nAChR activity and trafficking through the reduction of isoprenoids FPP and GGPP (Eckert et al., 2009), the hippocampal slices were co-treated with SV (10 μM) and farnesol (FOH, 2 μM) or geranylgeraniol (GGOH, 2 μM), which convert FPP and GGPP, for 2 h. The addition of FOH partially reduced the SV-increased *I*_ACh_ (*p* < 0.05, *n* = 8 cells/4 mice; **Figure 3A**), but failed to alter basal amplitude of *I*_ACh_ in control slices (*p* > 0.05, *n* = 8 cells/4 mice). By contrast, the application of GGOH had no effects on the basal amplitude of *I*_ACh_ and the SV-increased *I*_ACh_ (*p* > 0.05, *n* = 8 cells/4 mice). Notably, the SV-increased biotinylated α7nAChR protein (*p* < 0.01, *n* = 6 mice, 3–4 slices/mouse; **Figure 3C**) and SV-decreased cytoplasmic α7nAChR protein (*p* < 0.05, *n* = 6 mice; **Figure 3D**) were blocked by the addition of FOH, but not GGOH (*p* > 0.05, *n* = 6 mice).

The farnesylation of small GTPases is catalyzed by farnesyl transferase. To further determine whether the treatment with SV by the inhibition of farnesylation enhances the α7nAChR activity and trafficking, we examined the effects of the farnesyl transferase inhibitor FTI277 (FTI) on the α7nAChR activity and trafficking. As expected, the treatment with FTI (1 μM) for 2 h significantly increased *I*_ACh_ amplitude (*p* < 0.05, *n* = 8 cells/4 mice; **Figure 3B**) in a MLA-sensitive manner (*p* < 0.01, *n* = 8 cells/4 mice). Interestingly, the FTI-increased *I*_ACh_ was further enlarged by the addition of SV (*p* < 0.05, *n* = 8 cells/4 mice), although it was not affected by the addition of FOH (*p* > 0.05, *n* = 8 cells/4 mice). Similarly, the treatment with FTI increased the level of biotinylated α7nAChR protein (*p* < 0.01, *n* = 6 mice) with a concurrent decrease in cytoplasmic α7nAChR protein (*p* < 0.05, *n* = 6 mice). However, these parameters were not affected by the addition of SV (*p* > 0.05, *n* = 6 mice) or FOH (*p* > 0.05, *n* = 6 mice). These results indicate that SV through suppressing farnesylation of small GTPases enhances α7nAChR activity and surface localization, whereas the enhancing effect of FTI on α7nAChR activity is weaker than that of SV.

### Effects of SV on PKA, PKC, and CaMKII Phosphorylation

**FIGURE 4:**
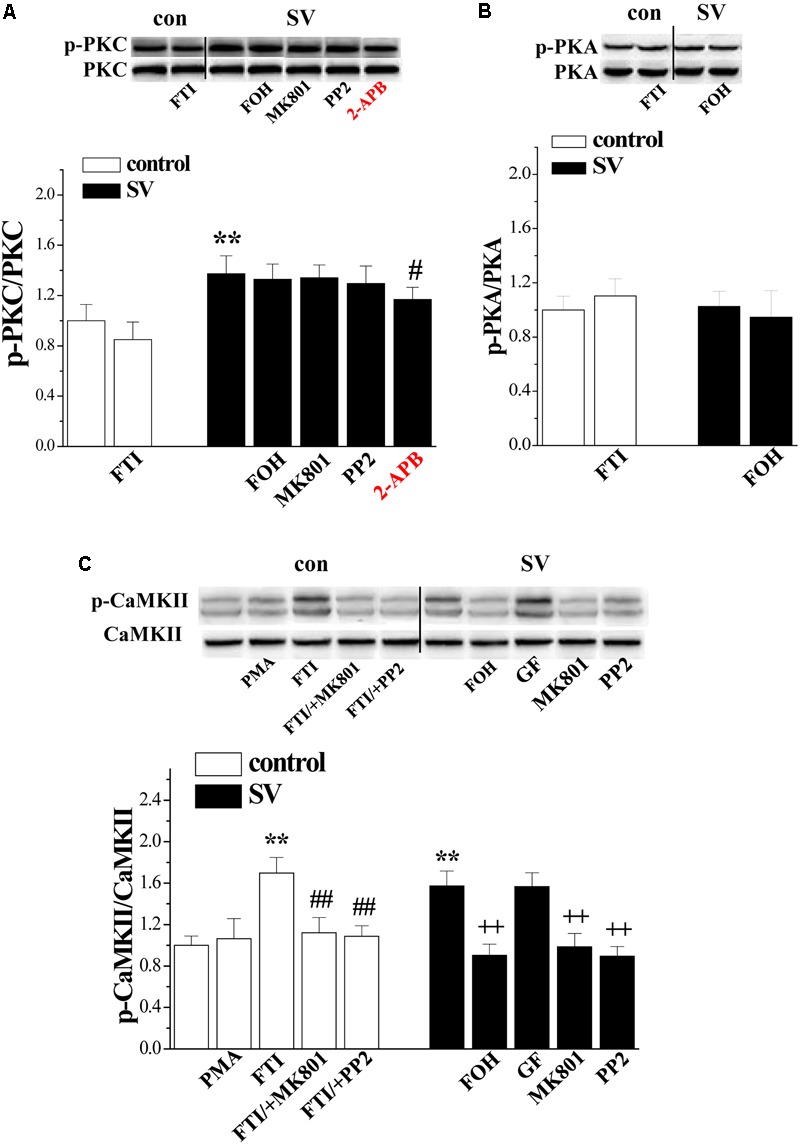
Simvastatin cascades PKC and CaMKII signaling pathways. **(A)** Bar graphs show the levels of phospho-PKC in FTI-treated slices, SV/+FOH-, SV/+MK801-, SV/+PP2- and SV/+2-APB-treated slices. ^∗∗^*p* < 0.01 vs. control slices; ^#^*p* < 0.05 vs. SV-treated slices. **(B)** Bar graphs show the levels of phospho-PKA in FTI-treated slices, SV/+FOH-treated slices. **(C)** Bars represent the levels of phospho-CaMKII in PMA- or FTI-treated slices, FTI/+MK801- or FTI/+PP2-treated slices, SV/+FOH-, SV/+GF-, SV/+MK801- and SV/+PP2-treated slices. ^∗∗^*P* < 0.01 vs. control slices; ^##^*p* < 0.01 vs. FTI-treated slices; ^++^*p* < 0.01 vs. SV-treated slices (two-way ANOVA).

A large body of evidence indicates that farnesylation status of small GTPases alters their interactions with intracellular molecules to regulate downstream effectors including PKC, PKA, and CaMKII (McTaggart, 2006). Therefore, we examined the effects of SV (10 μM) and FTI (1 μM) on the phosphorylation of PKC𝜀 (phospho-PKC𝜀), CaMKII (phospho-CaMKII), and PKA (phospho-PKA) (*n* = 6 mice per experimental group, 2 slices/mouse were treated). Notably, the treatment with SV elevated the levels of phospho-PKC𝜀 (*p* < 0.01; **Figure 4A**) and phospho-CaMKII (*p* < 0.01; **Figure 4C**), but not phospho-PKA (*p* > 0.05; **Figure 4B**). The SV-increased phospho-CaMKII (*p* < 0.01), but not phospho-PKC𝜀 (*p* > 0.05), was blocked by the treatment with FOH for 2 h. Interestingly, the treatment with FTI increased the level of phospho-CaMKII (*p* < 0.01) and failed to alter the level of phospho-PKA (*p* > 0.05). Although the FTI treatment had a tendency to reduce the phospho-PKC𝜀, the difference was not significant compared to the control slices (*p* > 0.05). The addition of the Src inhibitor PP2 (10 μM) or the NMDAr antagonist MK801 (10 μM) for 2 h abolished the SV- and FTI-induced increases in phospho-CaMKII (*p* < 0.01). However, the SV-increased phospho-PKC𝜀 was insensitive to PP2 (*P* > 0.05), MK801 (*P* > 0.05). Notably, the IP3R blocker 2-APB (75 μM) could partially prevent the SV-increased phospho-PKC𝜀 (*P* < 0.05). The co-treatment with the PKC inhibitor GF109203X (100 nM) failed to alter the SV-increased phospho-CaMKII (*p* > 0.05). The 2 h treatment with the PKC activator PMA (100 nM) had no effect on the phospho-CaMKII (*p* > 0.05). These results indicate that SV triggers the Src/NMDAr-dependent CaMKII signaling pathway through suppressing farnesylation of small GTPases. Meanwhile, the SV-increased phospho-PKC𝜀 was not associated with a decrease in FPP.

### Involvement of PKC/CaMKII in SV-Increased α7nAChR Activity and Trafficking

**FIGURE 5:**
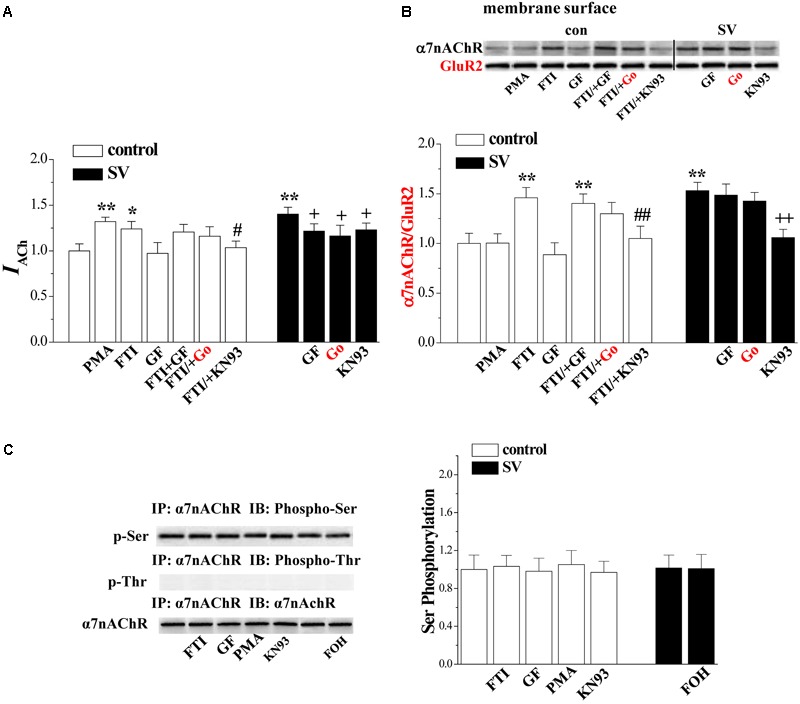
Involvement of PKC and CaMKII signlaing pathways in SV-enhanced α7nAChR activity and trafficking. **(A,B)** Bar graphs show the amplitude of *I*_ACh_ and the levels of biotinylated α7nAChR protein in PMA-, GF- or FTI-treated slices, FTI/+GF-, FTI/+Go- or FTI/+KN93-treated slices, SV/+GF-, SV/+Go- or SV/+KN93-treated slices. ^∗^*p* < 0.05 and ^∗∗^*p* < 0.01 vs. control slices; ^#^*p* < 0.05 and ^##^*p* < 0.01 vs. FTI-treated slices; ^+^*p* < 0.05 and ^++^*p* < 0.01 vs. SV-treated slices (two-way ANOVA). **(C)** Bars indicate the levels of phospho-α7nAChR in FTI-, GF-, PMA- or KN93-treated slices, SV/+FOH-treated slices.

The activity of α7nAChR is regulated by the PKC and CaMKII signaling pathways (Komal et al., 2015). To test the possibility, the hippocampal slices were co-treated with SV and the PKC inhibitors GF1092203X and Go6983 (10 nM) or the CaMKII inhibitor KN93 (3 μM). Interestingly, the treatment with either GF1092203X (*P* < 0.05, *n* = 8 cells/4 mice; **Figure 5A**) and Go6983 (*P* < 0.05, *n* = 8 cells/4 mice) or KN93 (*P* < 0.05, *n* = 8 cells/4 mice) partially suppressed the SV-increased *I*_ACh_ amplitude. By contrast, the SV-increased biotinylated α7nAChR protein was blocked by the addition of KN93 (*P* < 0.01, *n* = 6 mice; **Figure 5B**), but not GF1092203X (*P* > 0.05, *n* = 6 mice) or Go6983 (*P* > 0.05, *n* = 6 mice). In addition, the FTI-increased *I*_ACh_ amplitude (*P* < 0.05, *n* = 8 cells/4 mice) and biotinylated α7nAChR protein (*P* < 0.01, *n* = 6 mice) were completely blocked by the addition of KN93. The addition of GF1092203X or Go6983 had no effect on the FTI-increased *I*_ACh_ (*P* > 0.05, *n* = 8 cells/4 mice) and biotinylated α7nAChR protein (*P* > 0.05, *n* = 6 mice). In control slices, the PKC activation by PMA increased *I*_ACh_ amplitude (*P* < 0.01, *n* = 8 cells/ 4 mice), but failed to alter the level of biotinylated α7nAChR protein (*P* > 0.05, *n* = 6 mice). Furthermore, the application of GF1092203X alone had no effects on the *I*_ACh_ amplitude (*P* > 0.05, *n* = 8 cells/4 mice) or the biotinylated α7nAChR protein (*P* > 0.05, *n* = 6 mice). These results indicate that SV-increased α7nAChR activity depends on the activation of PKC and CaMKII, while SV- and FTI-increased α7nAChR trafficking are CaMKII-dependent.

Serine 365 in the M3-M4 cytoplasmic loop of the α7nAChR is a phosphorylation site for PKA (Moss et al., 1996). Phosphorylation of α7nAChR by PKC modulates the receptor channels (Huganir and Greengard, 1990). Based on this, we examined the phosphorylation of α7nAChR (phospho-α7nAChR) (*n* = 6 mice). The results showed that the treatment with SV (10 μM) or FTI (1 μM) for 2 h failed to alter the levels of phospho-α7nAChR (*p* > 0.05; **Figure 5C**). In addition, the treatment with GF109203X, PMA or KN93 did not affect the level of phospho-α7nAChR (*p* > 0.05).

## Discussion

The principal findings of this present study were that the acute treatment of hippocampal slices with SV enhanced the activity and trafficking of α7nAChRs on CA1 pyramidal cells. This conclusion is based on the following observations. (1) Treatment with SV induced a dose-dependent increase in *I*_ACh_ amplitude. (2) SV treatment caused a dose-dependent increase in α7nAChR protein on the cell membrane with a decrease in cytoplasmic α7nAChR protein level. (3) Removal of cholesterol and sphingomyelin from lipid rafts through acute cotreatment with statins and myriocin can slow the desensitization kinetics and increase the agonist affinity for α7nAChR (Colon-Saez and Yakel, 2011). The treatment with SV for 2 h did not alter the half-time (ms) of α7nAChR desensitization. The SV treatment increased the level of maximal α7nAChR response without the changes in EC_50_ and Hill coefficient of dose–response curve. The findings suggest that the SV treatment did not alter the affinity of α7nAChR. (4) The phosphorylation status of α7nAChR was not altered by treatment with SV.

Lovastatin is reported to be an inhibitor of Ras farnesylation (Laezza et al., 2008). Statin therapy causes a decrease in the isoprenoid FPP levels (Eckert et al., 2009). The SV-induced increase in *I*_ACh_ amplitude and trafficking of α7nAChRs were suppressed by the addition of FOH to increase the level of FPP. FPP is essential for the Ras farnesylation, a process catalyzed by prenyltransferase farnesyl transferase (Kho et al., 2004). Indeed, the treatment of FTI could increase the amplitude of *I*_ACh_, which was insensitive to FOH. The increase in *I*_ACh_ amplitude due to FTI treatment was further elevated by the addition of SV. Furthermore, the FTI treatment caused an increase in α7nAChR protein levels in the cellular membrane with a concurrent decrease in the cytoplasmic α7nAChR protein levels. The FTI-enhanced α7nAChR trafficking was not affected by the addition of SV or FOH. Like treatment with SV, the α7nAChR phosphorylation was not altered by the treatment with FTI. Therefore, it is highly likely that SV by reducing FPP suppresses the Ras farnesylation, which enhances the α7nAChR trafficking leading to the increase in α7nAChR activity.

The treatment of SV or FTI can enhance GluN2B and GluN2A phosphorylation via increasing Src phosphorylation (Chen et al., 2016b). We in this study observed that the SV treatment enhanced phosphorylation of CaMKII, which was blocked by the addition of FOH and mimicked by the FTI application. Moreover, SV- and FTI-enhanced phosphorylation of CaMKII was sensitive to the Src inhibitor PP2 or NMDAr antagonist MK801. Tyrosine phosphorylation of the NMDAr GluN2B and GluN2A subunits and NMDAr activity are higher in the hippocampus of H-Ras null mice than wild type mice (Manabe et al., 2000). Thus, it is conceivable that the treatment with SV via inhibition of Ras farnesylation enhances the Ca^2+^ influx of NMDAr leading to an increase in the CaMKII phosphorylation. There are conflicting reports describing that the CaMKII signaling is a possible upstream activator of Ras, because after NMDAr channels open CaMKII becomes activated briefly prior to Ras activation (Yasuda et al., 2006; Lee et al., 2009). Moreover, there is opposite results showing that the CaMKII phosphorylation inhibits the Ras activity (Chen et al., 1998). PKC potentiates NMDAr gating to enhance Ca^2+^ influx, which induces the CaMKII autophosphorylation (Wang and Kelly, 1995; Yan et al., 2011). However, our results did not support the idea, since the PKC inhibitors GF109203X and Go6983 failed to affect the SV- and FTI-enhanced phosphorylation of CaMKII.

The SV- and FTI-induced increases in biotinylated α7nAChR protein were blocked by the CaMKII inhibitor, the Src inhibitor or NMDAr antagonist. The inhibition of CaMKII abolished the FTI-increased α7nAChR activity, but partially prevented the SV-increased α7nAChR activity. The results give an indication that SV and FTI via elevation of CaMKII phosphorylation enhances the α7nAChR trafficking. Responses of α7nAChRs are enhanced by activating CaMKII (Kanno et al., 2012a) through stimulating α7nAChR trafficking or increasing receptor localization in the membrane (Derkach et al., 1999; Malinow and Malenka, 2002). Src family kinase has been reported to directly interact with the cytoplasmic loop of α7nAChR and phosphorylates the receptors at the membrane. The Src inhibition by PP2 increases the α7nAChR-mediated responses (Charpantier et al., 2005). Moreover, the tyrosine kinase inhibitor genistein causes the rapid delivery of α7nAChR to the membrane, which is not accompanied by detectable changes in receptor open probability (Cho et al., 2005).

Another critical finding in this study is that the phosphorylation of PKC was increased by the SV treatment, but this effect of SV was insensitive to the addition of FOH. The most common H-Ras mutation causes an irreversible decrease in PKC activity (Colapietro et al., 1993). PKC𝜀 is upregulated by oncogenic Ras activation (Dann et al., 2014). Similarly, the suppressed farnesylation of small GTPases by FTI had a tendency to reduce the PKC𝜀 phosphorylation. There is a potent activation of the kinase domain of PKCδ in response to atorvastatin in promyelocytic leukemia cells (Sassano et al., 2012). Acute treatment with atorvastatin has been reported to promote the Ca^2+^-mediated PKC activation (Pierno et al., 2009) through a mechanism involving release of Ca^2+^ from mitochondria and sarcoplasmic reticulum (Liantonio et al., 2007). Indeed, the SV-increased phospho-PKC𝜀 was partially prevented by the blocked of IP3R, rather than the NMDAr antagonist or the inhibition of Src. Further experiments are required to explore the mechanisms underlying SV-enhanced PKC phosphorylation.

**FIGURE 6:**
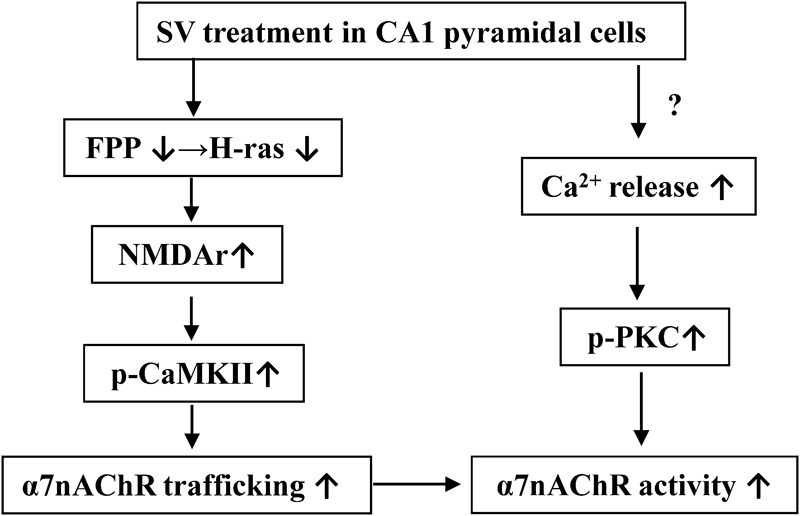
The hypothesis of molecular mechanisms underlying the SV-enhanced α7nAChR activity and trafficking. ↑: increase; ↓: decrease.

Several studies provide evidence to support the enhancing effects of PKC𝜀 on the α7nAChR activity (Miyamoto et al., 2003; Yamamoto et al., 2005). Although the α7nAChRs do not contain PKC phosphorylation sites (Moss et al., 1996), the activation of PKC potentiates activity of α7nAChRs (Nishizaki and Sumikawa, 1997). We observed that the PKC activator PMA increased α7nAChR activity, but had no effect on trafficking of α7nAChR. The SV-induced increase in *I*_ACh_ amplitude was reduced by a PKC inhibitor. The activation of PKC-𝜀 in the brain potentiates α7nAChRs responses to stimulate presynaptic glutamate release (Kanno et al., 2012c). Activation of PKC can enhance α7nAChR activity by increasing membrane surface localization of the receptor (Kanno et al., 2012b). However, we observed that SV- and FTI-increased α7nAChR trafficking are PKC-independent. Interestingly, we found that the treatment with SV at concentration of 1 μM could increase the *I*_ACh_ amplitude, while 5 μM SV was required for observable enhancement of α7nAChR trafficking. Overall, there are two possible mechanisms underlying the SV-increased α7nAChR activity, which are summarized in **Figure 6**. One is that SV through reduced FPP and Ras farnesylation can elevate the Src phosphorylation leading to increase in the Ca^2+^ influx of NMDAr, which enhances the α7nAChR trafficking via increasing CaMKII activity. The other is that SV through enhanced probably IP3R-mediated release of Ca^2+^ causes an increase in the PKC phosphorylation, which potentiates the α7nAChR activity.

Several studies have reported the reduction and dysfunction of α7nAChRs in the brains of AD patients(Kihara et al., 2004; Barrantes et al., 2010). Agonists of α7nAChR have been demonstrated to have beneficial effects on cognitive disorders in AD patients (Ren et al., 2007). Although the mechanism underlying SV-enhanced α7nAChR activity is not completely understood, these results in the present study can help in understanding the anti-dementia effects of treatment with statins in AD patients.

## Author Contributions

TC and LeC performed the electrophysiological experiments and all statistical analyes. YW and TZ undertook the immunoprecipitation and western blot analysis. BZ carried out the animal care. LiC and LZ carried out the experimental design and the preparation of the manuscript.

## Conflict of Interest Statement

The authors declare that the research was conducted in the absence of any commercial or financial relationships that could be construed as a potential conflict of interest.
